# Intracellular and Extracellular pH and Ca Are Bound to Control Mitosis in the Early Sea Urchin Embryo *via* ERK and MPF Activities

**DOI:** 10.1371/journal.pone.0066113

**Published:** 2013-06-13

**Authors:** Brigitte Ciapa, Laetitia Philippe

**Affiliations:** Centre de Neurosciences Paris-Sud (CNPS), CNRS UMR 8195 Université Paris XI, Orsay, France; Institute of Zoology, Chinese Academy of Sciences, China

## Abstract

Studies aiming to predict the impact on marine life of ocean acidification and of altered salinity have shown altered development in various species including sea urchins. We have analyzed how external Na, Ca, pH and bicarbonate control the first mitotic divisions of sea urchin embryos. Intracellular free Ca (Ca_i_) and pH (pH_i_) and the activities of the MAP kinase ERK and of MPF regulate mitosis in various types of cells including oocytes and early embryos. We found that intracellular acidification of fertilized eggs by Na-acetate induces a huge activation of ERK at time of mitosis. This also stops the cell cycle and leads to cell death, which can be bypassed by treatment with the MEK inhibitor U0126. Similar intracellular acidification induced in external medium containing low sodium or 5-(N-Methyl-N-isobutyl) amiloride, an inhibitor of the Na^+^/H^+^ exchanger, also stops the cell cycle and leads to cell death. In that case, an increase in Ca_i_ and in the phosphorylation of tyr-cdc2 occurs during mitosis, modifications that depend on external Ca. Our results indicate that the levels of pH_i_ and Ca_i_ determine accurate levels of Ptyr-Cdc2 and P-ERK capable of ensuring progression through the first mitotic cycles. These intracellular parameters rely on external Ca, Na and bicarbonate, alterations of which during climate changes could act synergistically to perturb the early marine life.

## Introduction

Studies aiming to predict the impact of increasing Carbon dioxide (CO_2_) emissions on marine life have shown a wide range of effects of acidification on echinoderms larval development rates, survival rates and body size [1], [2]. One can predict a crucial role of HCO3^−^ transporters to control pH_i_ of early sea urchin embryos. In point of fact, primary mesenchyme cells (PMCs) of sea urchin larva can compensate an induced intracellular acidosis *via* mechanisms that depend on external Na^+^ and HCO_3_^−^ [3], [4]. Altering the Na^+^/H^+^ exchanger and/or the external HCO_3_^−^concentration may then also lead to alterations of pH_i_ at earlier times of development, i.e. during the first mitotic divisions. Changes in pH_i_ occur during the cell cycle [5], and sea urchin eggs have been a pioneer model used in this domain of research. An increase in pH_i_ due to the activation of a Na^+^/H^+^ exchanger occurs after fertilization [6], and its inhibition stops the cell cycle [7], [8]. When pH_i_ is reduced after fertilization, DNA synthesis is stimulated but mitotic events are impaired [9]. The mechanisms that regulate pH_i_ and those that are responsible for the mitotic alterations during acidosis are not known. We hypothesized that alterations of pH_i_ could affect mitosis by modifying Ca_i_ levels as reported in somatic cells [10]. Acidosis can alter the activities of the Na^+^/Ca^++^ exchanger [11], Ca-ATPases of the plasma membrane [12] or the inositol trisphosphate-receptors (IP_3_R) of the endoplasmic reticulum (ER) [13] that all control Ca_i_ levels. Indeed, Ca_i_ changes were first detected during the first mitotic cycles in sea urchin embryos [14] and were then found in other types of cells [15]. Inhibition of the mitotic Ca_i_ transients arrests the first cell cycle, and a crucial role has then been conferred to Ca_i_ in the control of development of early embryos [14].

Progression in the cell cycle is highly regulated by complex mechanisms. Some of them involve kinases such as CDK (cyclin dependant kinases) or MAP kinases (Mitogen-activated protein kinases) and imply MPF (mitosis promoting factor) and ERK (extracellular regulated kinase), respectively [16], [17]. Ca controls MPF activity and therefore entry and exit of mitosis [18], [19]. Whether and how Ca_i_ controls ERK during early embryogenesis has not been studied in as much depth. In somatic cells, the MEK/MAPK cascade plays a major role in the regulation of distinct and even opposing processes, such as proliferation vs arrest of mitosis, or survival vs cell death [17]. In oocytes, this pathway is well known to regulate meiotic maturation and a high ERK activity stops the cell cycle before fertilization in matured oocytes [20], [21]. In all species, including sea urchin [22], [23], fertilization triggers inactivation of ERK. ERK is then slightly stimulated at first mitosis, in sea urchin as shown in our previous reports [22], [23] and in Xenopus [24], suggesting that ERK might control mitosis. A high ERK activity arrests the first mitotic cycle in Xenopus [25]. Suppression of ERK activity at fertilization is due to the stimulation of a Ca dependant MAPK phosphatase [26], but how Ca and ERK act on each other during mitosis is not well understood. We previously reported that artificial inactivation of ERK in unfertilized sea urchin eggs triggers mitosis entry by altering Ca_i_ levels [27]. Therefore, ERK might also control mitotic divisions by regulating Ca_i_ levels. Interestingly, inhibition of the MEK/ERK cascade has been reported by others to prevent the mitotic Ca_i_ transients and mitosis entry in another sea urchin species (*L. pictus*) [28]. However, the authors also detect an inactive ERK in unfertilized eggs, which contradicts the established view that down regulation of ERK is a consequence of fertilization in the animal kingdom [16], [20]. This prompted us to reinvestigate the role of ERK on Ca_i_ homeostasis at mitosis.

These data show various interactions between Ca_i_ and pH_i_, MPF and ERK activities, Cai and MPF, Cai and ERK but how they all interrelate has never been investigated. The present study shows that they depend on external Ca^++^, Na^+^ and bicarbonate and that they are tightly bound together to regulate the first mitotic divisions of the sea urchin embryo.

## Materials and Methods

### Handling of Gametes and Artificial Sea Water

Gametes of *Paracentrotus lividus* were collected, prepared and fertilized in artificial sea water (ASW, Reef Crystals Instant Ocean) as previously described [22], [23], [27]. Acidosis experiments were performed in ASW deprived of Na (470 mM Choline Cl, 27 mM MgCl2, 27 mM MgSO4, KHCO3 2 mM, 10 mM KCl, pH 8.0) containing 10 mM CaCl2 (0Na) or not (0Na0Ca), in ASW containing 50 µM 5-(N-Methyl-N-isobutyl amiloride (Am) or 50 mM NaAcetate, pH 6.7 (Ac), in ASW deprived of Ca (0Ca: 490 mM NaCl, 27 mM MgCl2, 27 mM MgSO4, 10 mM KCl, Na2HC03 2 mM, 1 mM EGTA, pH 8.0), in 0Na or Ac deprived of Ca (0Na0Ca and Ac0Ca, respectively). Effect of external HCO_3_^−^ was tested by removing it from these different media, thus giving 0HCO_3_, 0Na0HCO_3_, 0Na0Ca0HCO_3_. Eggs were fertilized in ASW and transferred 15–20 mins after sperm addition by five successive rinses into one of the acidifying ASW, where they were left to develop until the end of the experiment (1–4 hours).

### Inhibitors

These were dissolved as stock solutions in DMSO and added at a final concentration as followed: U0126 (Cell Signaling, 9903, 10 mM, 2 µM final), Emetine (Sigma, E2375, 50 mM, 100 µM final), Roscovitine (Sigma, R7772, 5 mM, 20 µM final), 5-(N-Methyl-N-isobutyl amiloride (Sigma, A4562, 2 mM, 50 µM final). Na/Ca inhibitors were Bepridil hydrochloride (BHC, Tocris 4117), SN6 (Tocris 2184) and KB-R7943 mesylate (KB, Sigma, R7493), in 50 mM stock solution and used at 10 µM final. Eggs were left to develop in these dilutions. A defined amount of DMSO was added to the egg culture as a control of all experiments.

### Microscopy, Ca_i_ and pH_i_ Imaging

Ca_i_ was measured as described previously [33] by using 10kDa-Fura-2 dextran (Molecular Probes) and shown as the ratio of fura-2 (340/380 nm excitation ratio). pH_i_ was measured following this same protocol, by using 5 mM BCECF (Molecular Probes) and by determining 440/480 excitation ratios. Images were taken with a Nikon D300S mounted on a microscope Eclipse TE300 equipped with a x20 plan fluor Nikon objective. In order to pool the data, Ca_i_ changes were calculated as R/Ro, where R is the ratio of fura-2 emissions obtained at 340 nm and 380 nm and Ro the ratio determined at time zero. Similarly, pH_i_ changes were calculated as R/Ro, where R is the ratio of BCECF emissions obtained at 440 nm and 480 nm. This gives a value of unfertilized eggs as 1. In this manner, results are presented as “Relative changes” in Ca_i_ or pH_i_. Discontinuous observations of sample of eggs were performed under a Nikon Eclipse TE300 equipped with a x20 plan Nikon objective.

### Western Blot Analysis

Preparation of egg samples, Western blotting and dilutions of all antibodies (all from Cell Signaling) were performed as previously described [22], [23], [27] with mouse anti-phospho-ERK 42/44 (Thr202/Tyr204), mouse anti-p44/42 ERK antibody, rabbit anti-Phospho-cdc2 (Tyr15) or rabbit anti- cdc2. Intensity of signals has been analysed using Image J and the ratios of P-ERK/ERK and Ptyr-cdc2/cdc2 calculated. Results are given relative to the ratios measured at time zero which was arbitrarily taken as 100.

## Results

### Ca_i_ and pH_i_ Levels during Mitosis Depend on the MEK/ERK Pathway and CDK Activity

**Figure 1:**
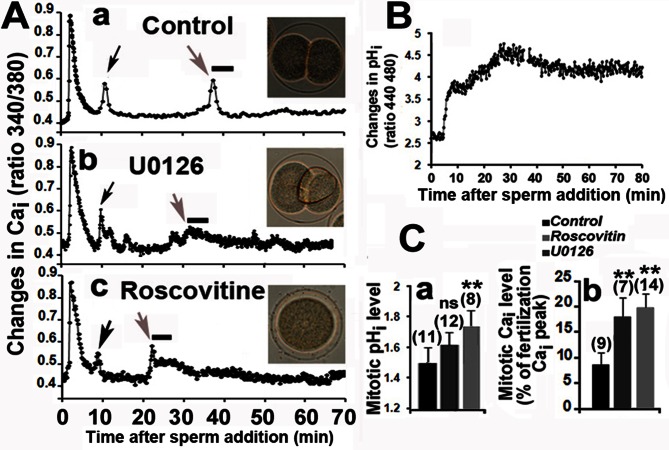
ERK and CDK activities control Ca_i_ and pH_i_ after fertilization. A. Time courses of Ca_i_ changes after fertilization in eggs treated or not (a) 10 mins after sperm addition (time zero) with 2 µM U0126 (b) or 20 µM Roscovitine (c). One typical recording is shown for each condition. Ca_i_ transients occur at time of pronuclear migration (black arrows) and at mitosis (grey arrow). Inset images taken 80 mins after fertilization show normal division in control eggs (a), mitotic alterations with U0126 (b) and absence of mitosis with Roscovitine (c). B. Time course of pH_i_ changes after fertilization of control eggs. C. Relative changes in pH_i_ (a) and Ca_i_ (b) levels in eggs treated or not (control) with 2 µM U0126 or 20 µM Roscovitine. The mean levels of pH_i_ recorded from 60 and 65 mins following sperm addition are expressed relative to that of unfertilized egg (arbitrarily taken as 1). Mean levels of Ca_i_ recorded during 5 mins at time of the mitotic peak (grey arrow and line in Fig. A), i.e. between 35–40 mins in control eggs, 30–35 mins in U0126 and 25–30 mins in Roscovitine treated eggs, are expressed as the percentage of the fertilization Ca_i_ peak arbitrarily taken as 100. The total number of eggs monitored is indicated for each condition (brackets). Values (mean +/− sem) are significantly higher than that of control eggs (**, student test, p<0.01) or not significantly different (ns).

Our recordings of Ca_i_ modifications after fertilization are similar to those frequently reported [29], showing a large transient Ca_i_ at fertilization followed by smaller Ca_i_ transients at the time of pronuclear migration and during mitosis (Fig. 1Aa). We previously reported that 2 µM U0126, the widely used MEK inhibitor, induced in unfertilized eggs a dephosphorylation of P-ERK and an increase in Ca_i_ level responsible for entry into mitosis [27]. Mitotic Ca_i_ changes occurred earlier and were significantly higher than those measured in control eggs when 2 µM U0126 were added 10 mins after fertilization, although these eggs divided (Fig. 1Ab), despite alterations of mitosis as expected [22]. Similar alterations in Ca_i_ mitotic levels were measured in fertilized eggs treated with 20 µM Roscovitine, an inhibitor of CDK that did not modify the P-ERK level (not shown) but blocks the cell cycle before mitosis by inhibiting MPF activity [30] and nuclear envelope breakdown (NEB) (Fig. 1Ac).

We next checked whether Ca_i_ and pH_i_ levels were correlated at time of mitosis. No significant changes during mitosis were reported by using dimethymoxazolidinedione (DMO) [31]. However, this weak acid takes at least 10 mins for equilibration in the egg, and the lack of synchrony of the population of eggs that was used for such experiments may mask variations that are small in time and intensity. On the contrary, recording of pH_i_ using microelectrodes showed a transient fall of pH_i_ prior to mitosis [32]. Fig. 1B shows a rapid and large increase in pH_i_ during the first 10 mins following fertilization that is due to the stimulation of a Na^+^/H^+^ exchange [6], [33]. This was followed by a 5 min slight drought, then pH_i_ increased again to reach its highest value 30 mins after fertilization, ie 5–10 mins before NEB. pH_i_ then slowly decreased until cytokinesis occurred but remained at a level higher than that of the unfertilized egg. Similar changes in pH_i_ were recorded when 2 µM U0126, 100 µM Emetine or 20 µM Roscovitine were added 10 mins after fertilization (not shown), although in the latter case pH_i_ reached during mitosis was significantly higher than in control eggs (Fig. 1Ca). This first set of data indicates that alterations of mitotic events can be associated with modifications of Ca_i_ (Fig. 1Cb) and pHi (Fig. 1Ca) levels.

### Ca_i_ Levels Control Mitotic Events

In order to test the impact of an aberrant increase in Ca_i_ at mitosis, 5 µM A23187 were added at that time. This triggered a transitory increase in Ca_i,_ a slight increase in pH_i_ during mitosis and deep alterations of mitotic events although eggs divided (Fig. S1). Refilling of intracellular Ca_i_ stores after fertilization can take at least 30 min [34]. This process depends on Ca transporters of the plasma membrane and therefore relies on external Ca availability [15]. Transfer of fertilized eggs 10 mins after fertilization into 0Ca did not modify the time course of Ca_i_ and of pH_i_ during the first cycle and eggs divided normally (Fig. S2). Therefore, and at first sight, external Ca does not seem to be necessary for Ca_i_ and pH_i_ regulation and progression of into the first mitotic cycle.

### External Na and Ca control Ca_i_ and pH_i_ Levels and Determine Cell Cycle Progression

**Figure 2:**
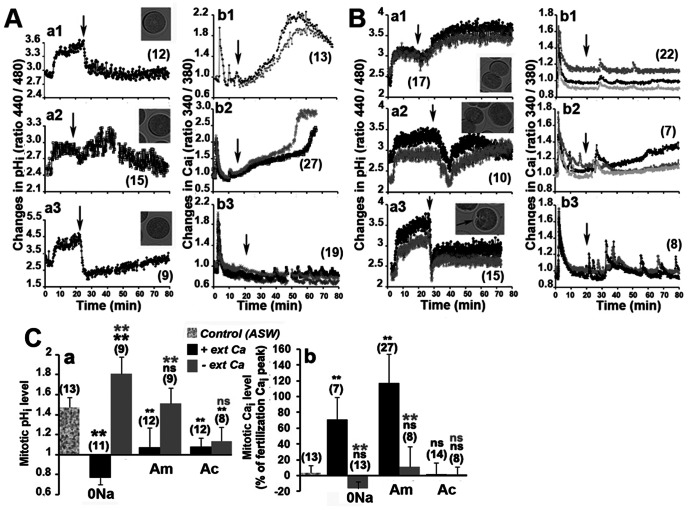
Changes in Ca_i_ and pH_i_ during mitosis depend on external Ca^++^ and Na^+^. Eggs were transferred in the different media after fertilization (arrow). Two examples representative of n measurements (number in brackets) are shown in each condition. Variations in pH_i_ (a), evolution of Ca_i_ (b) and images of eggs taken 70 min after fertilization (insets in a). A. Transfer in 0Na (1), Am (2) or Ac (3). B. Effect of additional absence of external Ca after transfer in 0Na0Ca (1), Am0Ca (2) or Ac0Ca (3). **C**. Mean levels of pH_i_ (a) and Ca_i_ (b) changes recorded from 60–65 mins after sperm addition in Figs. A and B. pH_i_ changes (a) are expressed relative to that of unfertilized egg (arbitrarily taken as 1) while Ca_i_ changes (b) are expressed as the percentage of the fertilization Ca_i_ peak arbitrarily taken as 100 (zero is the unfertilized level). Values (means +/− sem) obtained in the absence of external Ca are significantly different (student test, p<0.01, two black stars) or not (ns, black letters) from control. Values obtained in the absence of Ca are significantly different (p<0.01, two grey stars) or not (ns, grey letters) from those obtained in the presence of Ca. Number of eggs is indicated for each condition (brackets).

It has been shown that arrest of division occurs when fertilization is performed in Ac or 0Na, or in ASW containing amiloride, an inhibitor of the Na^+^/H^+^ exchanger. However, the fertilization pH_i_ signal and all early events that are related to it are cancelled in these experiments [6]. We then transferred eggs into these different acidifying ASW 20 mins after fertilization, i.e after these events. Acidification was induced after transfer of eggs in 0Na (Fig. 2Aa1), in ASW containing 50 µM 5-(N-Methyl-N-isobutyl) amiloride (Am), which is 20–100 times more potent and specific than amiloride [35], [36] (Fig. 2Aa2) or in Ac (Fig. 2Aa3). In all conditions, pH_i_ came back to the level of the unfertilized egg at time of mitosis of the control eggs, although time for acidification was longer in Am, perhaps due to the time required for the chemical to bind the Na^+^/H^+^ exchanger [35]. Control eggs divided normally (not shown), but none of the eggs transferred in 0Na or Am entered mitosis as no NEB was observed (Fig. 2Aa1 and a2) while a few eggs showed NEB in Ac (Fig. 2Aa3). Transfer in 0Na (Fig. 2Ab1) or in Am (Fig. 2Ab2) led to a huge increase in Ca_i_ that could reach, 60 mins after fertilization, a level that was higher than that measured during the fertilization transient Ca_i_ signal. Regular small Ca_i_ transients, which looked like Ca_i_ oscillations, were detected at a constant frequency after transfer in Ac (Fig. 2Ab3).

These alterations in Ca_i_ could be due to an entry of Ca from the external medium. We then performed the same experiments in the absence of external Ca, i.e. in 0Na0Ca, Am0Ca or Ac0Ca. After a drop in pH_i_ that was measured in all cases at the time of rinse, the pH_i_ of eggs transferred in 0Na0Ca (Fig. 2Ba1) and Am0Ca (Fig. 2Ba2) increased again to reach a value similar to that of 60 min or 30 min fertilized control eggs (Fig. 1C), respectively, while that of eggs rinsed with Ac0Ca stabilized at a value slightly higher than that of unfertilized eggs (Fig. 2Ba3). Clearly, Ca_i_ did not accumulate in 0Na0Ca (Fig. 2Bb1) or Am0Ca (Fig. 2Bb2), although the latter condition triggered the appearance of a peak of Ca_i_ at mitosis larger than that usually detected at that time in control eggs (Fig. 1Aa). Ca_i_ oscillations were still detected in Ac0Ca but they occurred with a greater amplitude and lower frequency than those recorded in the presence of external Ca (Fig. 2Ab3). Eggs divided normally in 0Na0Ca and in Am0Ca (Fig. 2Ba1 and Fig. 2Ba2) while a few eggs showed constrictions and attempts at division in Ac0Ca (Fig. 2Ba3). All results are summarized in Fig. 2C. Absence of Na, or presence of 5-(N-Methyl-N-isobutyl) amiloride, induces acidosis (Fig. 2Ca) and a large increase in Ca_i_ level (Fig. 2Cb) by mechanisms that rely on the presence of external Ca, while Ac triggers acidosis in the presence or not of external Ca (Fig. 2Ca) and does not increase the Ca_i_ level, whether external Ca is present or not (Fig. 2Cb). These results suggest that Na^+^/H^+^ and mechanisms relying on external Ca are closely linked to regulate Ca_i_, pH_i_ and mitosis.

### Acidosis Leads to Modifications in P-ERK and PTyr-cdc2

**Figure 3:**
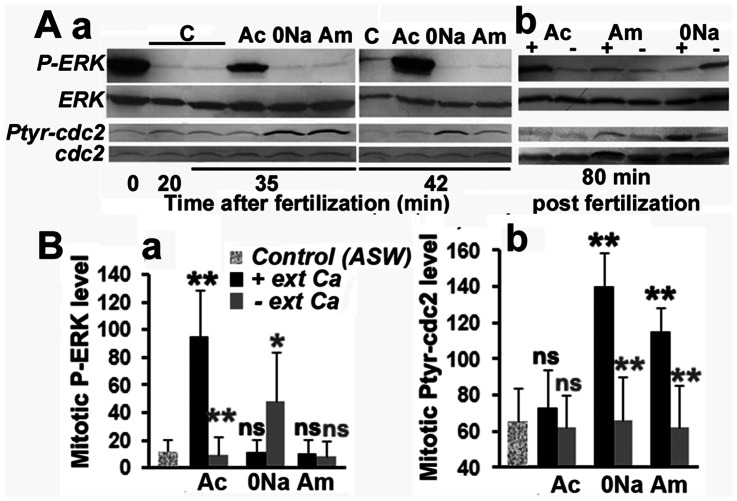
Ca_i_ and pH_i_ define P-ERK and Ptyr-cdc2 levels at mitosis. A. Changes in Ptyr-cdc2 and P-ERK levels induced in fertilized eggs transferred or not (control, C) 20 mins after fertilization in Ac, 0Na or Am in the presence (+) or not (–) of external Ca. Total ERK and cdc2 do not vary during the experiment. B: Compiled assessment of P-ERK (a) and Ptyr-cdc2 (b) signals obtained at time of mitosis as visualized in A, and from three experiments that gave similar results. Values (means +/− sem) are significantly different (student test, p<0.01, two black stars or p<0.05, one black star) or not (ns, black letters) from control. Comparison between those obtained in the absence or presence of external Ca is also given (grey stars and letters).

We tested whether these modifications in Ca_i_ and pH_i_ induced in acidifying ASW could affect the levels of Ptyr-cdc2 and P-ERK. Results are displayed in Fig. 3A and summarized in Fig. 3B. Unfertilized eggs contain a highly phosphorylated ERK that was rapidly inactivated during the first 20 mins after fertilization (Fig. 3Aa). A slight reactivation was detected 42 mins after fertilization, i.e. during mitosis, in control eggs but not in 0Na and Am, while a substantial increase in P-ERK levels, corresponding to that detected in unfertilized eggs, was seen at that time in Ac (Fig. 3Aa). Although levels of Ptyr-cdc2 were low at mitosis in control eggs and in Ac, they were markedly increased in 0Na and in Am (Fig. 3Aa). Therefore, a low level of P-ERK seems to correspond to high levels of PTyr-cdc2 at mitosis (in 0Na and Am), while high levels in P-ERK parallels low levels of PTyr-cdc2 (in Ac). The increase in P-ERK seen in Ac and in PTyr-cdc2 levels in 0Na and Am did not occur in absence of external Ca, i.e. in Ac0Ca, 0Na0Ca and Am0Ca, respectively (Fig. 3Ab and Fig. 3B). These results suggest that the arrest of the cell cycle induced by Ac could be due to altered stimulation of P-ERK while that induced by 0Na and Am would be due to a high level of PTyr-cdc2 which corresponds to a reduced MPF activation.

### P-ERK Level Controls Cell Cycle Progression and Survival After Acidosis

**Figure 4:**
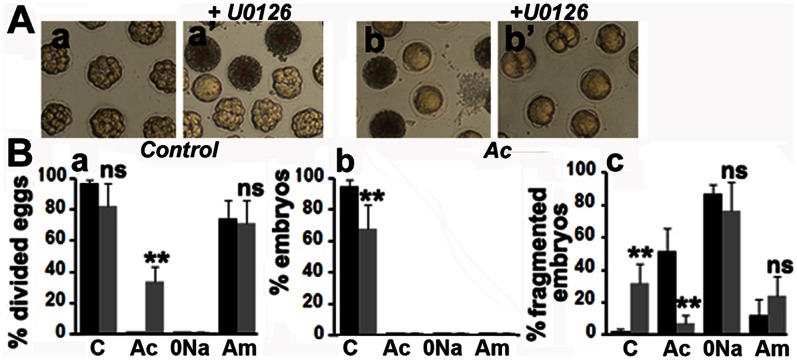
P-ERK activity controls mitosis and survival of fertilized eggs. A. Images taken 3 hours after fertilization of eggs transferred or not (control) 25 mins after fertilization in Ac containing or not 2 µM U0126. B. The experiment shown in A was performed three times and the data obtained pooled. Eggs were also transferred in 0Na or Am. % of divided eggs (a), embryos (b) or fragmented eggs (c) were determined 3 hours after fertilization (filled histograms) except for divided control eggs that were counted 80 mins after sperm addition (hatched histograms). 42–78 total embryos were counted in each condition. Values (means +/− sem) obtained in the presence of U0126 (grey histogram) are significantly different (student test, p<0.01, two stars) or not (ns) to those obtained without this drug (black histogram).

In order to check whether the block of mitosis in Ac could be bypassed by suppressing ERK reactivation, fertilized eggs were transferred 20 mins after fertilization in Ac containing or not 2 µM U0126 where they were allowed to develop. The effect of U0126 was also tested in 0Na and Am. P-ERK was not detected up to 3 hours after fertilization in any embryos treated with U0126 (not shown). Control eggs attained the 16-cell stage (Fig. 4Aa) while a significant proportion of embryos treated with U0126 were dead or abnormal (Fig. 4Aa’). None of the eggs in Ac divided and a low % was fragmented (Fig. 4Ab), whilst treatment of these eggs with U0126 induced a small percentage of division and reduced fragmentation (Fig. 4Ab’). Eggs in 0Na were fragmented and 70% of embryos in Am were at the 2-cell stage in the presence or not of U0126 (not shown). Similar results were obtained when eggs were allowed to develop after the first mitosis and then transferred to the different acidifying ASW containing U0126 or not (not shown). Compilation of data indicate that U0126 significantly increased cell division (Fig. 4Ba), although not leading to embryonic development (Fig. 4Bb), and decreased fragmentation (Fig. 4Bc) of embryos in Ac, but had no effect on embryos in Am or 0Na. This set of data suggests that Ca_i_ and pH_i_ vary in parallel with ERK and MPF activities to control both cell division and survival.

### External HCO_3_^−^ Controls Ca_i_ and pH_i_ Levels

**Figure 5:**
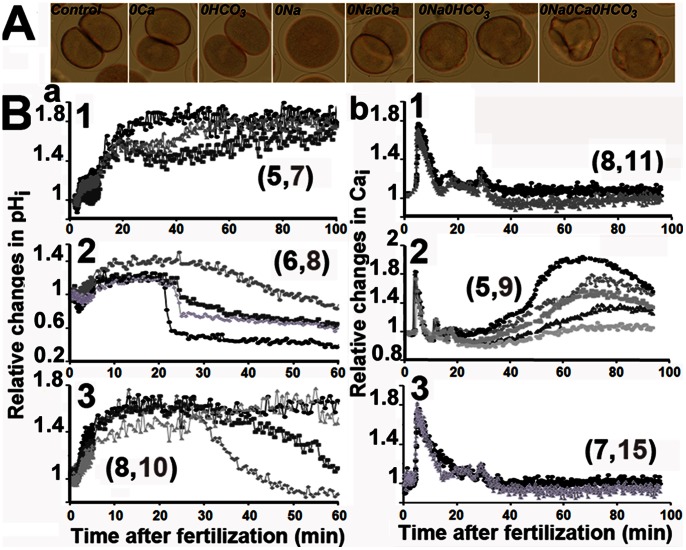
Ca_i_ and pH_i_ depend on the presence of external HCO3^–^. A. Observation 80 mins after fertilization. Eggs develop in ASW (Control) or are transferred 20 mins after fertilization into ASW deprived of Ca (0Ca), HCO_3_^−^ (0HCO_3_) or of Na^+^ and Ca^++^ (0Na0Ca). B. Time course after fertilization of pH_i_ (a) and Ca_i_ (b) in ASW, 0Na and 0Na0Ca in the presence or not of HCO_3_^−^. Examples of results obtained in absence of HCO3-, i.e. in 0HCO_3_ (1), 0Na0HCO_3_ (2) and 0Na0Ca0HC0_3_ (3), are depicted in different grey colours and representative of n determinations (grey number in brackets). In each condition, one typical control experiment performed with the same population of eggs in the presence of HCO_3_^−^, i.e. in ASW (1), 0Na (2) and 0Na0Ca (3) is shown in black curve and representative of n determinations (black number in brackets).

Acidosis induced in low external Na could depend on external HCO_3_^−^ as has been described in PMCs [4]. Removing external HCO_3_^−^ from ASW containing normal high Na concentration did not affect division (Fig. 5A, 0HCO_3_). As reported above, fertilized eggs did not enter mitosis after transfer in 0Na (Fig. 5A), but a few eggs showed NEB and cellular constrictions when external HCO_3_^−^ was also absent (Fig. 5A, 0Na0 HCO_3_). Division of fertilized eggs that occurred in 0Na0Ca as expected (Fig. 5A) was inhibited when external HCO3^−^ was also removed (Fig. 5A, 0Na0Ca0HCO_3_). Morphologic observations were correlated with pH_i_ (Fig. 5B a) and Ca_i_ changes (Fig. 5Bb). No clear difference in both ionic events was noticed between eggs dividing in ASW containing HCO3^−^ or not (Fig. 5Ba1 and Fig. 5Bb1). On the contrary, absence of HCO_3_^−^ compensated the intracellular acidosis (Fig. 5Ba 2) that is induced in absence of external Na and reduced the large increase in Ca_i_ (Fig. 5Bb2) with an intensity that varied with the egg. This could explain why a few eggs progressed into the cell cycle, showing NEB and constrictions (Fig. 5A). Finally, absence of external HCO_3_ in 0Na0Ca induced acidosis (Fig. 5Ba3) with an intensity that also varied with the eggs but did not modify the Ca_i_ changes (Fig. 5Bb3). Altogether, these data suggest that Ca_i_ and pH_i_ both rely on mechanisms dependant on external Na, Ca and HCO_3_^−^.

### Ca_i_ and pH_i_ Levels Depend on Na/Ca at Mitosis

**Figure 6:**
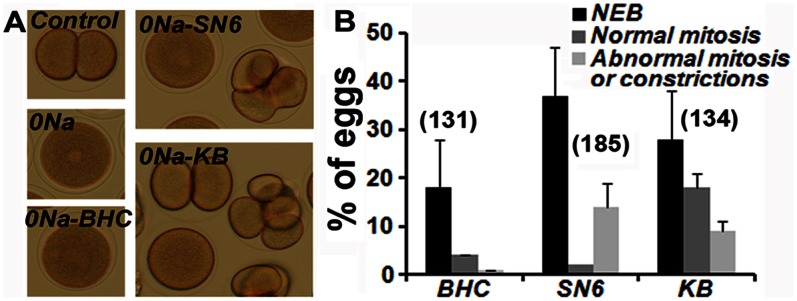
Na/Ca inhibitors reduce inhibition of mitosis induced in the absence of external Na. Eggs were let to develop in ASW (Control) or transferred 20 min after fertilization in 0Na containing or not (0Na) the different inhibitors, BHC, SN6 or KB. Observation 80 min after fertilization (A) and compiled assessment of results (means +/− sem) from 3 experiments that gave similar results (B). Total number of eggs counted in each condition is indicated in brackets in B.

Our results suggest that acidification occurring in the absence of external Na^+^ could be coupled to Ca^++^ entry. We therefore investigated the role of Na^+^/Ca^2+^-exchange on pH_i_ and Ca_i_ regulation by using Bepridil hydrochloride (BHC), a calcium channel-blocking chemical that is also a mitoKATP channel opener/sarcKATP channel blocker [37], SN6, a more selective Na^+^/Ca^2+^-exchange (NCX) inhibitor that has some affinity for mACh [38], and KB-R7943 mesylate (KB), an inhibitor of the reverse mode of the Na^+^/Ca^2+^ exchanger that also inhibits the mitochondrial Ca^2+^ uniporter [39]. None of these chemicals significantly altered cell cycle progression of fertilized eggs allowed to develop in ASW (not shown) but all of them reduced the inhibition rate of mitosis entry and division of eggs transferred in 0Na (Fig. 6). A significant proportion of eggs showed NEB in the presence of 10 µM BHC (Fig. 6A, 0Na-BHC), although none of them could progress further to division (Fig. 6B). A higher rate of eggs undergoing NEB was obtained with 10 µM SN6 and a few eggs showed constrictions but none of them divided (Fig. 6A, 0Na-SN6 and Fig. 6B). Finally, KB used at 10 µM was the more potent of these chemicals to reverse the effect of low external Na^+^ since significant percentages of eggs showed NEB, constrictions or even cleavage-like division of control eggs (Fig. 6A, 0Na-KB and Fig. 6B). These results suggest that Ca influx is coupled to Na^+^ efflux to regulate mitotic division.

## Discussion

The present study shows that Ca_i_ and pH_i_ closely interact to control the first mitotic cycles of the sea urchin embryo. Their levels at mitosis rely on the Na^+^/H^+^ and Na^+^/Ca^++^ exchangers and on the presence of extracellular HCO3^−^, which sets up appropriate grades of PTyr-Cdc2 and P-ERK capable of ensuring progression through the cell cycle. To our knowledge, this is the first report showing that Ca_i_ and pH_i_, ERK and CDK activities exert a tight reciprocal control on each other to regulate mitosis. This should help to understand the panel of alterations that is seen when one only of these events is modified. For example, they could explain the reduced rates of mitotic divisions that occur after a drop of pH to 7.0 [40].

### Correlation pH_i_/Ca_i_

The first rise in pH_i_ took a longer time than previously described in *P. lividus* [33] and *S. purpuratus* [31], although the kinetics are compatible with those reported in *L. pictus* [32]. The short drop of pH_i_ in *P. lividus* that occurred 10 mins after fertilization could be due to mechanisms of the plasma memabrane that pumps out Ca^++^ against H^++^ [11], [12] to decrease the high Ca_i_ level reached after the fertilization Ca_i_ signal. The drop in pH_i_ occurring at NEB correlates with results obtained on egg homogenates of *S. purpuratus* [41] and may be related to mitosis entry since pH_i_ remained at high levels in Roscovitine treated eggs where mitosis is blocked. pH_i_ remained at levels higher at mitosis than during interphase, which corroborates data reported in mouse embryos [42] or in proliferating fibroblasts [43]. It is peculiar to note that mitotic arrest, after inhibition of CDK activity or of protein synthesis with Emetine (not shown), led to alkalosis and consequent apoptosis (not shown) and not to acidosis which is often reported to be at the origin of cell death [44]. However, an increase in apoptosis during alkaline stress has also been proposed [45]. Embryos developed normally at ext pH 6.8, which did not modify Ca_i_ and pH_i_ (not shown). As reported by Johnson and Epel [31], low external pH needed to be associated with acetate addition to decrease pH_i_ and to arrest cell cycle progression. This suggests that sea urchin embryos are capable of highly regulating their pH_i_ in response to external pH fluctuations.

It is unlikely that the variations in Ca_i_ seen after pH_i_ changes within the range reported are due to effects of pH on the properties of fura-2, the calcium probe that we used here [46]. Ca_i_ measurements might have been underestimated at acidic pH [47], but if that were the case, increases in Ca_i_ seen in 0Na or in Am would be even larger than the values we have reported.

It was assumed that regulation of pH_i_ in the sea urchin egg mainly depends on the Na^+^/H^+^ exchanger [31], [33]. We found that neither acidification nor increase in Ca_i_ occurred when both external Ca^++^ and external Na^+^ were missing. Furthermore, pH_i_ was not sustained at high levels when external HCO3^−^ was also removed, which can explain why embryos did not divide in these conditions. These results indicate a role of other mechanisms besides Na^+^/H^+^ such as Na^+^/HCO_3_^−^ and Na^+^/Ca^++^ [48], [49] to maintain the alkaline pH_i_ of the fertilized eggs. The role of Na^+^/Ca^++^ is reinforced by the effect of SN6 and KB that are inhibitors of this exchanger [38], [39]. We cannot exclude a role of mACh that is also inhibited by SN6 [38] and that could control mitosis in the sea urchin embryo [50]. The presence of mechanisms that regulate Ca_i_ and sense extracellular Ca^++^ as Na^+^/Ca^++^ or Ca-ATPase is reinforced by the appearance of Ca_i_ oscillations that appear after transfer in Ac and occur with an enhanced amplitude in the absence of external Ca. As a point of fact, Ca_i_ oscillations are highly regulated by these Ca sensors [51] and can be generated after acidosis with acetate via the activity of the Na/H exchanger [52].

### Interaction pH_i_/Ca_i_ and Cell Cycle Regulation

Levels of Ca_i_ reached during mitosis were increased after inhibition of the MEK/ERK cascade with U0126. These results are compatible with our previous observations in unfertilized eggs that enter mitosis and where sensitivity of IP_3_-R to IP_3_ is increased after inactivation of ERK [27]. Our results contradict those obtained by others with 100 µM U0126 [28]. However, this substantial amount of U0126 might non-specifically act on kinases other than MEK [53]. Altogether, alterations of mitosis observed in U0126 treated eggs or after A23187 addition indicate that mitotic Ca_i_ transients must be controlled in space, time and amplitude for normal mitosis.

The arrest of cell cycle in Ac could be due to the strong reactivation of ERK at mitosis as has been described in vertebrates [25], [54]. This hypothesis is reinforced by the fact that preventing ERK reactivation allows a few embryos to divide. ERK might have been stimulated by the Ca_i_ oscillations generated in Ac. Indeed, Ca_i_ oscillations and strong ERK stimulation occur at the same time during oocyte maturation, but to our knowledge no correlation between the two events has ever been proven and the role of these Ca_i_ signals in the control of oocyte maturation is rather controversial [55].

Activities of ERK and MPF seem to be opposite during the first mitotic cycles, MPF activity being high when ERK is low. Inactivation of MPF in 0Na or in Am might be the cause of cell cycle arrest. This corroborates other data showing that acidic conditions inhibit dephosphorylation of Ptyr-cdc2 [30]. The high level of Ca_i_ reached in these conditions may alter cdc25 activity [18], [19] but could also be toxic and impair other mechanisms.

Finally, increased proliferation and embryo development was associated with survival for embryos transferred in Ac and treated with U0126. These results are similar to those found in hepatocytes where the MEK/ERK pathway either blocks proliferation after sustained activation, or allows multiple cell cycles, improving survival after transient activation [56].

### Conclusion

Since levels of Ca_i_ and pH_i_ rely on external Ca and Na, they could be altered with increased atmospheric CO_2_ concentration and global warming [57]. However, we are aware that climate change will not induce the drastic changes in sea water that we have induced in the present experiments. Larval growth is only slowed down and no direct impact on morphology or calcification has been reported when embryos are exposed to acidosis that do not exceed 7.25 [58]. However, consequences may be worse if acidification is associated with another change of the medium. For example, drop of salinity can occur in coastal sea of arctic zones where global warming causes the sea ice to melt. In this regard, rates of sea urchin fertilization and development are reduced when salinity is less than 25°/oo [59]. It would be of value to test whether small alterations in several climatic variables act synergistically and exacerbate a developmental stress.

## Supporting Information

Figure S1 Effect of absence of external Ca on Cai and pHi of fertilized eggs. Fertilized eggs were transferred into ASW-0Ca 10 mins after fertilization. A: Time course of Cai and pHi changes. Absence of external Ca did not modify Cai levels recorded during the first mitotic cycle (Fig. Aa) and eggs divided normally (Fig. Ab). Although pHi stopped rising during 10–15 mins at the time of transfer into ASW-0Ca, the kinetics of pHi changes until cleavage (Fig. Ac) was very similar to that measured in control eggs (Fig. 1Ca). B. Compiled assessment of Cai (a) and pHi (b) shown in A. The mean levels of Cai and pHi recorded from 60–65 mins following sperm addition were calculated and expressed as a percentage of fertilization Ca peak (see legend of Fig. 1B) or as relative change (see legend of Fig. 1Cb). Cai level of eggs dividing in ASW0Ca returns to unfertilized level as control eggs, (mean +/− sem) (a) and pHi is not significantly altered in 0Ca. The total number of eggs monitored is indicated for each condition (brackets).
(TIF)

Figure S2 Effect of absence of external Ca on Cai and pHi of fertilized eggs. Fertilized eggs were transferred into ASW-0Ca 10 mins after fertilization. A: Time course of Cai and pHi changes. Absence of external Ca did not modify Cai levels recorded during the first mitotic cycle (Fig. Aa) and eggs divided normally (Fig. Ab). Although pHi stopped rising during 10–15 mins at the time of transfer into ASW-0Ca, the kinetics of pHi changes until cleavage (Fig. Ac) was very similar to that measured in control eggs (Fig. 1Ca). B. Compiled assessment of Cai (a) and pHi (b) shown in A. The mean levels of Cai and pHi recorded from 60–65 mins following sperm addition were calculated and expressed as a percentage of fertilization Ca peak (see legend of Fig. 1B) or as relative change (see legend of Fig. 1Cb). Cai level of eggs dividing in ASW0Ca returns to unfertilized level as control eggs, (mean +/− sem) (a) and pHi is not significantly altered in 0Ca. The total number of eggs monitored is indicated for each condition (brackets).
(TIF)
