# Supplementary figures and images for: Intracellular and Extracellular pH and Ca Are Bound to Control Mitosis in the Early Sea Urchin Embryo via ERK and MPF Activities
Source: PLoS One. 2013 Jun 13;8(6):e66113. doi: 10.1371/journal.pone.0066113 (PMC3681939; doi:10.1371/journal.pone.0066113)

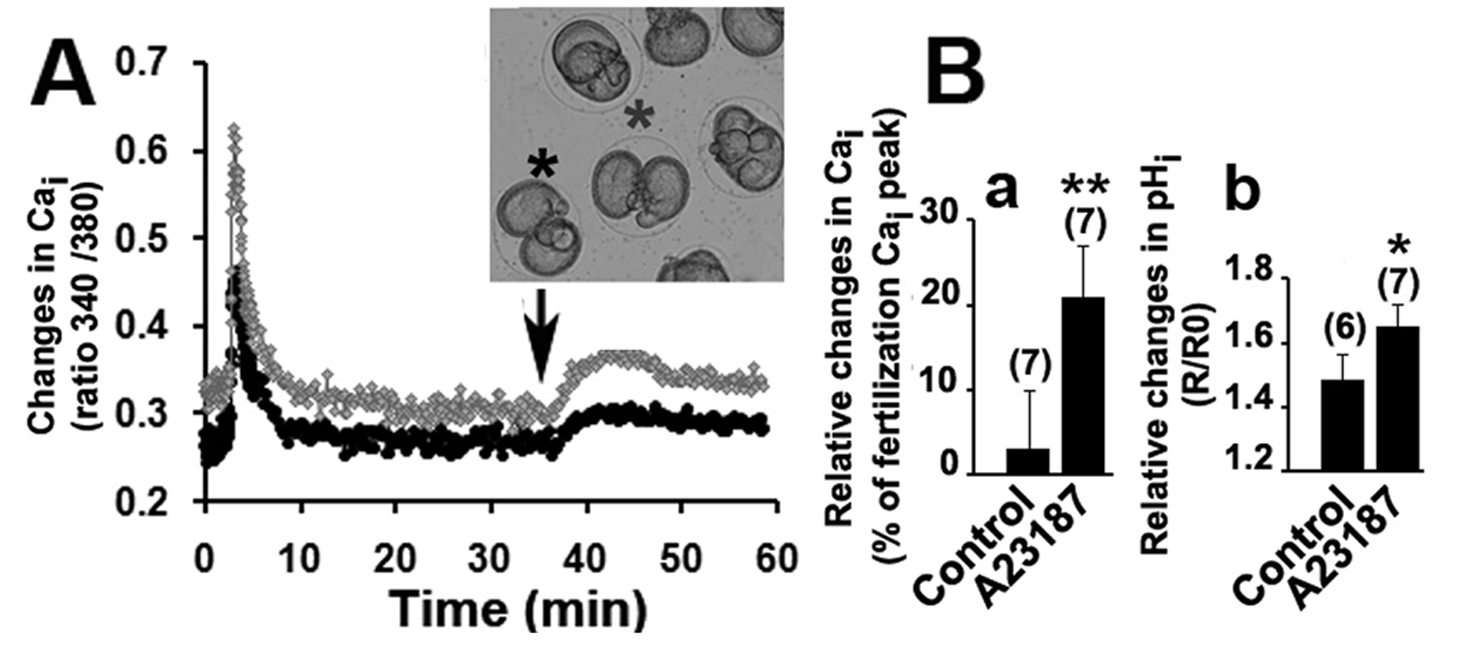

Supplement: Figure S1 — Effect of absence of external Ca on Cai and pHi of fertilized eggs. Fertilized eggs were transferred into ASW-0Ca 10 mins after fertilization. A: Time course of Cai and pHi changes. Absence of external Ca did not modify Cai levels recorded during the first mitotic cycle (Fig. Aa) and eggs divided normally (Fig. Ab). Although pHi stopped rising during 10–15 mins at the time of transfer into ASW-0Ca, the kinetics of pHi changes until cleavage (Fig. Ac) was very similar to that measured in control eggs (Fig. 1Ca). B. Compiled assessment of Cai (a) and pHi (b) shown in A. The mean levels of Cai and pHi recorded from 60–65 mins following sperm addition were calculated and expressed as a percentage of fertilization Ca peak (see legend of Fig. 1B) or as relative change (see legend of Fig. 1Cb). Cai level of eggs dividing in ASW0Ca returns to unfertilized level as control eggs, (mean +/− sem) (a) and pHi is not significantly altered in 0Ca. The total number of eggs monitored is indicated for each condition (brackets). (TIF) [file pone.0066113.s001.tif]

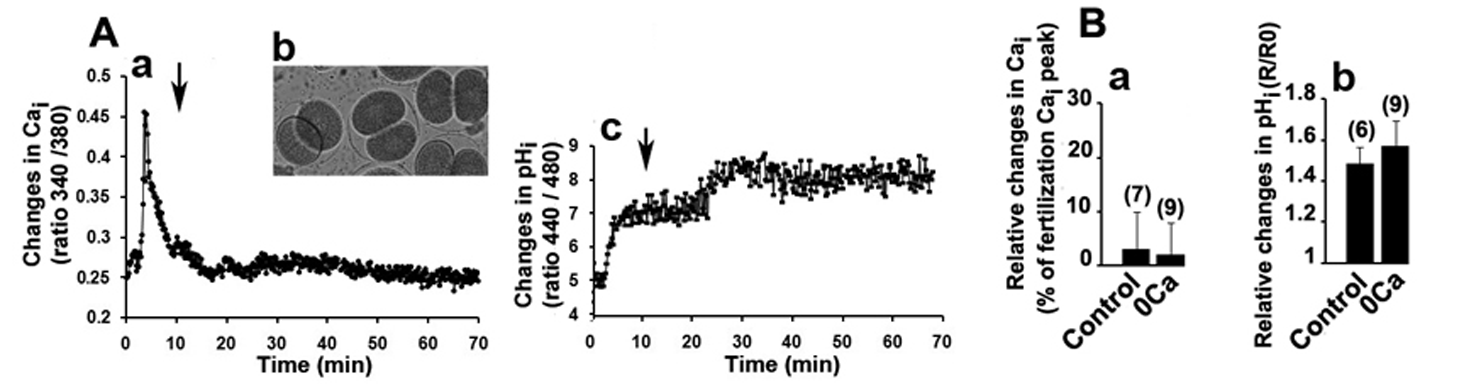

Supplement: Figure S2 — Effect of absence of external Ca on Cai and pHi of fertilized eggs. Fertilized eggs were transferred into ASW-0Ca 10 mins after fertilization. A: Time course of Cai and pHi changes. Absence of external Ca did not modify Cai levels recorded during the first mitotic cycle (Fig. Aa) and eggs divided normally (Fig. Ab). Although pHi stopped rising during 10–15 mins at the time of transfer into ASW-0Ca, the kinetics of pHi changes until cleavage (Fig. Ac) was very similar to that measured in control eggs (Fig. 1Ca). B. Compiled assessment of Cai (a) and pHi (b) shown in A. The mean levels of Cai and pHi recorded from 60–65 mins following sperm addition were calculated and expressed as a percentage of fertilization Ca peak (see legend of Fig. 1B) or as relative change (see legend of Fig. 1Cb). Cai level of eggs dividing in ASW0Ca returns to unfertilized level as control eggs, (mean +/− sem) (a) and pHi is not significantly altered in 0Ca. The total number of eggs monitored is indicated for each condition (brackets). (TIF) [file pone.0066113.s002.tif]
